# Psychosocial maturity and institutional confinement from ages 15–22: a longitudinal reciprocal model

**DOI:** 10.3389/frcha.2026.1809393

**Published:** 2026-06-10

**Authors:** Frances R. Chen, Donia Chiciu, Hawi Geleto

**Affiliations:** Department of Criminal Justice and Criminology, Georgia State University, Atlanta, GA, United States

**Keywords:** adolescents and young adults, correctional exposure, desistance, psychosocial maturity, random intercept cross-lagged panel model, temperance, perspective, responsibility

## Abstract

**Objectives:**

Psychosocial maturity develops rapidly during adolescence and early adulthood and is closely linked to delinquency and justice system involvement. Although incarceration and psychosocial immaturity have each been studied, they are rarely examined together within a single developmental framework, and modeling only one direction may inflate estimated effects. Moreover, psychosocial maturity is typically treated as a global construct, despite meaningful variation across its core components.

**Methods:**

We analyzed data from the longitudinal Pathways to Desistance study (*N* = 1,336), which follows serious adolescent offenders over time. Using Random Intercept Cross-Lagged Panel Models, we assessed bidirectional associations between psychosocial maturity and correctional time. Psychosocial maturity was assessed via three core components: temperance, responsibility, and perspective.

**Results:**

Lower psychosocial maturity predicted greater subsequent correctional time, and greater correctional time predicted slower-than-expected gains in psychosocial maturity. Standardized within-person effects were small but consistent across ages. Component-level analyses indicated that reciprocal associations were driven primarily by temperance.

**Conclusions:**

Initial incarceration driven by lower psychosocial maturity levels may foster heightened institutional contact that further disrupts normative developmental processes, particularly self-regulation. These findings support both importation and deprivation perspectives and provide empirical support for Moffitt's ensnarement hypothesis. Findings highlight the importance of jointly modeling developmental vulnerability and institutional exposure and underscore the role of self-regulatory capacities in justice system involvement. Developmental implications and juvenile justice policy are discussed.

## Introduction

Adolescence and early adulthood are critical periods of psychological and neurological development that shape long-term behavioral, emotional, and social trajectories. One particularly salient developmental construct during this time is psychosocial maturity, a set of evolving capacities including impulse control, future orientation, autonomy, and resistance to peer influence. These traits not only support adaptive functioning but are also strongly associated with desistance from crime. Yet many youths involved in the justice system remain caught in cycles of incarceration, raising questions about how institutional settings influence or disrupt normative development.

Much of the existing literature assumes a unidirectional process, treating incarceration as an exogenous event that impairs psychosocial development. This approach risks conflating institutional deprivation with selection processes, as youth with lower psychosocial maturity have been shown to experience deeper and more prolonged justice system involvement. Without modeling both directions simultaneously, estimates of institutional effects may be inflated by unmodeled reverse pathways. Moreover, psychosocial maturity comprises distinct components, temperance, responsibility, and perspective, that differ in developmental timing and susceptibility to environmental influence. Disaggregating these components allows for more precise identification of which aspects of psychosocial development are most closely linked to justice system involvement.

To address these issues, we employ a Random Intercept Cross-Lagged Panel Model (RI-CLPM), which separates stable between-person differences from within-person change over time. Using longitudinal data from serious adolescent offenders followed from ages 15–22, we examine reciprocal associations between psychosocial maturity, its core components, and correctional time to clarify developmental processes during a critical period.

### The impact of incarceration on youth development

Incarceration during adolescence and early adulthood has been consistently linked to a range of long-term disadvantages. When compared to the general population, youth that are imprisoned, particularly at age 14 or younger, experience worsening functional limitations preceding the deterioration of general health into adulthood (1). Incarceration also has immediate detrimental effects on economic stability (e.g., income, employment), although these effects are found to be part of a broader continuum of disadvantage that starts prior to incarceration (2). Beyond physical health, economic, and social functioning, early incarceration has been linked to long-term detrimental effects on mental health. Meta-analyses and systematic reviews show that compared to non-incarcerated youth, detained youth exhibit significantly higher rates of psychiatric disorders, including depression and posttraumatic stress disorder (3, 4). These mental health disparities highlight the acute psychological burdens carried by justice-involved youth.

Among these outcomes, psychosocial maturity stands out as both developmentally sensitive and behaviorally consequential. Psychosocial maturity is broadly examined as the ability to develop and maintain the social and moral obligation to navigate challenges throughout life (5). Psychosocial maturity encompasses a suite of capacities, such as self-regulation, empathy, and independent decision-making, that typically strengthen during adolescence through increased executive function and brain maturation (6, 7). Steinberg and Cauffman (5) conceptualize psychosocial maturity through three interrelated components: temperance (the ability to regulate impulsive and aggressive behavior), perspective (consideration of long-term consequences of one's actions and contemplation of the impact on themselves and others), and responsibility (autonomy and resistance to peer pressure).

Unlike many other outcomes, psychosocial maturity is not only an indicator of well-being, but also a key predictor of behavioral trajectories, including patterns of persistence or desistance from crime (8). As such, it serves as both a *developmental marker* and a *behavioral mechanism*, bridging what youth experience and how they act. Correctional environments may pose a unique threat to the normative development of psychosocial maturity. These settings often curtail autonomy, restrict meaningful social interaction, and impose highly regimented routines, thereby depriving adolescents of critical developmental experiences (66). Developmental models and empirical evidence suggest that chronic exposure to adversity can disrupt the maturation of self-regulatory and executive functioning capacities, constraining the development of agency and prolonging transitions from adolescence to adulthood (9, 10).

Empirical research supports this view. Dmitrieva et al. (11), using fixed-effects models with data from the Pathways to Desistance study, found that more time spent in institutional settings was associated with smaller gains in global psychosocial maturity, temperance, and responsibility in the short term. However, the impact of incarceration on psychosocial maturity was short-lived and not cumulative, because no sustained differences were identified over the 7-year period, as indicated by the lack of relationship between total confinement time and net change in psychosocial maturity. Monahan et al. (12), also using Pathways data, observed that exposure to violence corresponded with stagnation in impulse control and decision-making. While Monahan and colleagues did not examine confinement directly, their findings are relevant given that exposure to violence is a common experience in institutional settings (13, 14), suggesting that the conditions of confinement may indirectly contribute to developmental stagnation.

Complementing these findings, Thomas et al. (15) conducted a 16-year longitudinal study using a separate sample from the Northwestern Juvenile Project. Although their definition of psychosocial maturity was conceptualized differently within the Pathways dataset, they included related constructs such as emotional regulation and interpersonal functioning. Importantly, their multilevel models accounted for a wide range of preexisting characteristics, including baseline mental health, socioeconomic status, and offense severity, and still found that longer incarceration periods and confinement in more severe facilities (e.g., prisons) were associated with poorer psychosocial outcomes in adulthood. This existing body of research underscores various accounts of incarceration's direct role in delaying psychosocial maturation such that detained youths are guided toward maladaptive developmental tracks as a result.

These findings are further bolstered by insights from developmental neuroscience, which characterize adolescence as a sensitive period during which environmental conditions can exert powerful and enduring effects on brain architecture and behavior, particularly in the maturation of prefrontal regulatory systems and socioemotional circuitry (16, 17). In essence, incarceration may inhibit growth in precisely those faculties, such as self-regulation, autonomy, future orientation, and perspective-taking that are essential for successful reintegration and long-term desistance from crime. Research on institutional settings, including child welfare placements, suggests that highly controlled, low-autonomy environments can lead to enduring impairments in psychosocial functioning (18). Thus, evidence from related domains lends converging support for the view that placement in restrictive environments (e.g., correctional facilities) hinder critical dimensions of psychosocial maturity by constraining opportunities for normative developmental experiences.

### Psychosocial maturity as a predictor of incarceration and its implications

A large and well-established body of research demonstrates that psychosocial maturity is a robust predictor of delinquency, persistence in offending, and justice system involvement. Adolescents and young adults with lower levels of impulse control, weaker future orientation, and heightened susceptibility to peer influence are at elevated risk for engaging in criminal behavior and other high-risk activities (19, 20, 67). Across adolescence and early adulthood, longitudinal evidence further shows that psychosocial maturity is tightly linked to desistance, with lower maturity associated with persistent offending and growth in maturity accompanying eventual desistance (8, 21, 22, 68).

These patterns align with longstanding theoretical frameworks. General Theory of Crime identifies low self-control, a core component of psychosocial immaturity, as a fundamental cause of criminal behavior (23). Likewise, Moffitt's developmental taxonomy (10, 24) differentiates between “life-course persistent” offenders, whose behaviors are rooted in enduring psychological and neurodevelopmental deficits, and “adolescence-limited” offenders, whose delinquency tends to wane as psychosocial maturity develops. Both theories emphasize that developmental immaturity plays a central role in shaping the onset and persistence of criminal behavior.

Beyond predicting offending *per se*, psychosocial immaturity also affects how youth interact with the legal system. Theoretical and empirical research suggests that lower psychosocial maturity not only increases the likelihood of offending but may also compromise a young person's ability to effectively navigate legal processes, thereby compounding their risk of incarceration. Scott et al. (25) provide a conceptual framework for understanding how developmental immaturity can undermine legal decision-making. Supporting this, adolescents with lower judgment capacity may struggle to comprehend legal rights, assess plea options, and make decisions that serve their long-term interests, vulnerabilities that heighten the risk of deeper justice system involvement (26). Thus, psychosocial immaturity operates not only as a risk factor for crime but also as a mechanism shaping how youth are processed within the justice system.

Importantly, the existence of this pathway has critical implications for how the developmental consequences of incarceration are interpreted. When psychosocial maturity predicts subsequent incarceration, models that estimate only the effect of incarceration on development implicitly treat institutional exposure as exogenous. In doing so, they risk conflating developmental suppression caused by incarceration with selection processes driven by preexisting psychosocial vulnerability. As a result, one-directional models may overstate the magnitude of incarceration's developmental impact by capitalizing on associations that plausibly operate in both directions. This does not imply that institutional environments are benign or inconsequential; rather, it highlights the possibility that some portion of the observed association reflects who becomes incarcerated in the first place, not solely what incarceration does to development.

Recognizing psychosocial maturity as both a predictor and a potential outcome of incarceration therefore shifts the analytic focus from a unidirectional causal narrative to a dynamic developmental process. From this perspective, incarceration may suppress psychosocial growth, but the magnitude of this effect cannot be fully understood without accounting for the fact that lower psychosocial maturity also increases exposure to institutional environments. Disentangling these reciprocal processes is essential for accurately estimating institutional harm, identifying developmentally sensitive intervention points, and avoiding attribution errors that over- or understate the role of incarceration in shaping adolescent development.

### Disaggregating psychosocial maturity: differential pathways to incarceration

Psychosocial maturity is a multidimensional construct encompassing distinct but interrelated developmental capacities (5). Although often examined as a global index, its core components—temperance, responsibility, and perspective—may differ meaningfully in both their predictive relationships with incarceration and their susceptibility to institutional influence. Theoretical and empirical work suggests that these components operate through distinct developmental mechanisms, raising the possibility that reciprocal dynamics with incarceration are not uniform across domains.

A central contribution of the present study is to move beyond global measures of psychosocial maturity by examining these components separately within a reciprocal, within-person framework. Disaggregating psychosocial maturity allows us to identify which specific developmental capacities are most consequential for selection into incarceration, which are most vulnerable to suppression during institutional exposure, and whether these processes operate symmetrically across domains. In doing so, this approach clarifies whether observed associations between incarceration and psychosocial maturity reflect broad developmental disruption or are driven by particular facets of psychosocial functioning.

### Temperance: self-regulation and behavioral control

Temperance, reflecting impulse control and the suppression of aggressive responses, represents the component of psychosocial maturity most closely tied to immediate self-regulatory processes, shaping how individuals manage impulses and comply with rules in structured institutional environments (5, 23). From a developmental perspective, temperance is behaviorally proximal, shaping how individuals regulate emotions and inhibit impulses in real time, particularly under conditions of stress. Extensive research links deficits in self-regulation to delinquency, violent behavior, and persistent offending (23, 27, 28). Youths low in temperance are more likely to act reactively, violate rules, and struggle with emotional regulation, characteristics that increase both the likelihood of arrest and the probability of incarceration rather than diversion.

The development of temperance depends on repeated opportunities to practice impulse control, manage emotions, and learn from the consequences of self-directed behavior. These regulatory capacities are shaped through experiences that require individuals to exercise restraint under conditions that provide feedback and manageable challenges. Correctional environments, however, are often characterized by high stress, constant surveillance, and limited opportunities for autonomous regulation. Rather than fostering self-regulatory growth, such settings may reduce opportunities to practice impulse control and instead reinforce reactive or defensive coping strategies (11, 12).

As a result, temperance is one that is most likely to have a reciprocal process with correctional time: deficits in self-regulation increase the likelihood of justice system involvement and incarceration, while exposure to correctional environments may further suppress the development of regulatory capacities essential for behavioral control and desistance. This bidirectional dynamic underscores the importance of examining temperance separately from other components of psychosocial maturity and modeling its relationship with incarceration as an evolving developmental process rather than a unidirectional effect.

#### Responsibility: autonomy, self-reliance, and resistance to peer influence

Responsibility captures the development of autonomy, self-reliance, and resistance to peer pressure—capacities that reflect internalized self-governance rather than immediate behavioral control (5). Lower responsibility has been linked to delinquency and continued justice involvement, primarily through heightened peer influence and reduced ownership of decision-making (22, 29). Youths low in responsibility are more likely to remain embedded in criminogenic peer networks, less likely to disengage from antisocial social contexts, and slower to adopt prosocial roles associated with desistance (8, 21).

The implications of responsibility for time spent in correctional facilities are therefore less straightforward and likely to be indirect. Rather than precipitating confinement through impulsive or rule-breaking behavior, responsibility may shape how youth navigate justice system involvement over time, including their engagement with diversion programs and community supervision. To the extent that lower responsibility is associated with difficulties in autonomous decision-making or sustained compliance, it may contribute to cumulative justice system exposure through escalation processes such as revocations or placement failures.

From a developmental perspective, responsibility reflects the internalization of self-governance rather than momentary behavioral control. Its development is grounded in identity formation, role-taking, and repeated opportunities to make autonomous decisions and experience their consequences (5, 30, 31). These processes typically unfold through increasing independence, sustained social roles, and autonomy-supportive contexts, particularly during adolescence and early adulthood (32, 33).

Because responsibility develops through the gradual internalization of norms and identity-based self-governance rather than through moment-to-moment regulatory practice, it may be less sensitive to short-term institutional exposure than other components of psychosocial maturity. Correctional environments, which emphasize surveillance and compliance, offer few opportunities for autonomous decision-making or identity-relevant role-taking. However, the absence of these opportunities is more likely to *stall* the development of responsibility than to actively suppress it. Unlike self-regulatory capacities such as temperance, which are continuously taxed by stress and threat and therefore vulnerable to degradation under adverse conditions, responsibility may remain relatively stable during incarceration, neither substantially increasing nor declining. Consequently, responsibility may function more strongly as a predictor of incarceration than as an outcome shaped by it, suggesting an asymmetric developmental relationship.

#### Perspective: future orientation and consideration of consequences

Perspective encompasses future orientation and the capacity to consider the long-term consequences of one's actions for oneself and others. Extensive research links limited future orientation to delinquency, substance use, and criminal persistence, as youth who discount the future or prioritize immediate rewards are more likely to engage in risky and illegal behavior (34–36). Compared to temperance and responsibility, however, perspective represents a more distal, cognitive dimension of psychosocial maturity. Thus, perspective may not play a direct role in determining justice system responses such as confinement. Unlike deficits in temperance, which can precipitate immediate rule violations, or lower responsibility, which may indirectly compromise compliance and system navigation, limited future orientation does not necessarily interfere with day-to-day behavioral regulation or supervision demands (37). As a result, perspective may only weakly predict incarceration, if any, operating primarily through its influence on broader offending patterns rather than through mechanisms that directly increase correctional exposure.

Recent theoretical work emphasizes that short-term mindsets may not reflect developmental deficits *per se*, but rather adaptive responses to unstable, unpredictable, or resource-scarce environments (38). For justice-involved youth who face chronic unpredictability, limited resources, and restricted access to long-term rewards, prioritizing immediate outcomes may be a rational and functional orientation. Correctional environments, which emphasize short-term compliance and offer few opportunities for meaningful goal pursuit or future planning, are unlikely to disrupt—or substantially promote—changes in this cognitive orientation. Unlike temperance, which is continuously taxed by stress, perspective may persist largely unchanged during institutional exposure. Together, we predict that perspective may have weak or null associations with correctional time.

### The current study

The present study examines reciprocal associations between psychosocial maturity and correctional time during adolescence and early adulthood using longitudinal data from serious adolescent offenders. Rather than treating incarceration solely as a cause or consequence of psychosocial immaturity, we integrate importation and deprivation frameworks by explicitly modeling bidirectional within-person processes.

In addition to evaluating global psychosocial maturity, we disaggregate maturity into its core components to assess whether distinct developmental capacities differ in their reciprocal associations with correctional time. This component-level approach allows us to determine whether behaviorally proximal capacities, such as self-regulation, are more dynamically linked to justice system involvement than more distal, identity- or cognition-based capacities. By capturing bidirectional processes at both global and component levels across ages 15–22, the present study clarifies how psychosocial development and justice system involvement co-evolve during a sensitive developmental period, with implications for the timing and targets of developmentally informed intervention.

A further contribution of this study lies in its analytic approach. Prior longitudinal research has often relied on fixed-effects or related models to examine the developmental consequences of incarceration. While fixed-effects models effectively control for all time-invariant individual characteristics and isolate within-person change in outcomes, they do not explicitly model the stable trait component of time-varying predictors such as correctional exposure. As a result, variation in incarceration may reflect a combination of enduring between-person differences (e.g., overall propensity toward confinement) and dynamic within-person fluctuations, potentially obscuring how stable vulnerabilities and short-term deviations jointly shape developmental processes.

By contrast, Random Intercept Cross-Lagged Panel Models (RI-CLPM) explicitly decompose both psychosocial maturity and correctional time into between-person (trait-like) and within-person (state-like) components. This decomposition allows cross-lagged paths to be interpreted as pure within-person associations, that is, capturing whether deviations from an individual's expected developmental trajectory predict subsequent deviations in correctional exposure, and vice versa, while simultaneously modeling stable between-person differences.

From a developmental psychopathology perspective, understanding whether psychosocial immaturity is merely a precursor to incarceration or is itself altered by institutional exposure has direct implications for the timing and targets of intervention. Identifying which components of psychosocial maturity are most developmentally sensitive to correctional environments can inform both prevention efforts and the design of developmentally responsive institutional practices.

## Materials and methods

### Data and participants

Data were drawn from the Pathway to Desistance project. The Pathways to Desistance Project included 1,354 adolescents who were found guilty of a serious offense from Philadelphia County (Pennsylvania, *N* = 700) and Maricopa County in Arizona (Phoenix, *N* = 654) (39). The participants were 14–17 years old at the time of their committing offense, and these offenses were primarily felonies with some exceptions for misdemeanor offenses such as property offenses, sexual assault, or weapons offenses (40). Participants completed follow-up interviews for seven years (41). Specifically, follow-up interviews occurred 6, 12, 18, 24, 30, 36, 48, 60, 72, and 84 months after the baseline. The study's participation rate at initial enrollment was 67%. The response rate of the enrolled participants was very high over the 7 years follow up ranging from 84% to 93%.

The current analyses included only 1,336 of enrolled participants because 18 of them did not have data regarding their institutional experience in any follow-up periods. Male adolescents comprised 86.40% of the sample (*N* = 1,154). The sample was diverse in ethnicity, with 40.90% self-identified as black, 33.80% Hispanic, 20.50% white, and 4.72% other.

### Measures

#### Psychosocial maturity

Psychosocial maturity score was calculated drawing from four different inventories, the Weinberger Adjustment Inventory [WAI; (42)], the Future Orientation Inventory [FOI; (43)], Psychosocial Maturity Inventory [PSMI; (44)] and the Resistance to Peer Influence measure [RPI; (29)]. Psychosocial maturity scores were calculated by averaging temperance, perspective and responsibility scores, each of which are outlined below.

*Temperance* was assessed using the impulse control and suppression of aggression subscales from the WAI, designed to assess an individual's level of social-emotional adjustment within the context of external constraints. The WAI included three subscales, impulse control (8 items; e.g., “I say the first thing that comes into my mind without thinking enough about it”), suppression of aggression (7 items; e.g., “People who get me angry better watch out”), and consideration of others (7 items; e.g., “Doing things to help other people is more important to me than almost anything else”). Participants rated how well each statement described their behavior over the past six months using a 5-point Likert scale (1 = *False* to 5 = *True)*. To enable direct scoring and comparison with other measures of psychosocial maturity, the 5-point WAI scale was rescaled to a 4-point scale while preserving the relative spacing between points. This linear rescaling harmonized scale ranges across measures without altering the underlying distributional properties or relative differences among participants.

Importantly, scores were not standardized within age (e.g., wave-specific z-scoring). Because the Random Intercept Cross-Lagged Panel Model (RI-CLPM) decomposes stable between-person differences from within-person fluctuations over time, preserving the original scale metric is necessary to retain meaningful developmental variation. Wave-specific standardization would remove age-level mean differences and constrain between-person variance, thereby distorting the growth structure and variance partitioning central to RI-CLPM estimation. Maintaining the raw metric allows both mean-level developmental change and trait-like stability to be estimated directly within the model.

Average scores were calculated for the impulse control subscale when at least 6 items were completed, and for suppression of aggression when at least 5 items were completed. The *Temperance* score was the mean of these two subscales, and higher scores indicate greater temperance.

*Perspective* was assessed using the consideration of others subscale in WAI and the total score from FOI. Average scores were calculated for the consideration of others subscale in WAI when at least 5 items were completed. The FOI assesses individuals' attitude, goal, and expectation about the future using items from the Life Orientation Task (45), the Zimbardo Time Perspective Scale (46), and the Consideration of Future Consequences Scale (47). Participants rated from 1 to 4 (1 = *Never True* to 4 = *Always True*) the degree to which each statement reflects how they usually are (e.g., “I will keep working at difficult, boring tasks if I know they will help me get ahead later”). Although 15 items were on the FOI, only 8 items were used to form the total score (details can be found on the Pathways to Desistance website: http://www.pathwaysstudy.pitt.edu/codebook/foi-sb.html). Average scores were calculated when at least 6 items were completed. The *Perspective* score was the mean of these two scales. Higher scores indicate greater perspective.

*Responsibility* was assessed using the self-reliance subscale in PSMI and RPI. The PSMI contains three 10-item subscales, self-reliance (e.g., “Luck decides most things that happen to me”), identity (e.g., “I change the way I feel and act so often that I sometimes wonder who the ‘real’ me is”), and work orientation (e.g., “I hate to admit it, but I give up on my work when things go wrong”). Participants rated each item on a 4-point Likert scale (1 = *Strongly Disagree* to 4 = *Strongly Agree*). Average scores of self-reliance were calculated when at least 8 items were completed. The RPI is a 10-sequence measure that assesses how much weight adolescents put in the opinions of their peers. For each sequence, participants were first asked to choose the scenario that most closely reflects their behavior out of the two conflicting scenarios (e.g., “Some people go along with their friends just to keep their friends happy” and “Other people refuse to go along with what their friends want to do, even though they know it will make their friends unhappy”), and then to rate the degree to which the statement is accurate (i.e., “sort of true” or “really true”). Based on the scenario chosen and the rating, each sequence will get a score ranging from 1 to 4. The average score of RPI was calculated from these 10 dimensions. The *Responsibility* score was the mean of these two scales. Higher scores indicate greater responsibility.

#### Correctional facility stays

Participants were asked questions inquiring about the different settings for services they received across three services sectors: social services, juvenile justice, and the mental health system in the recall period. Setting types include drug/alcohol unit, psychiatric care, residential treatment center or group home, foster care, detention/prison/jail, and shelters. The total number of days spent in correctional facilities, including jails, prisons, or detention centers, was used in this study, because it reflects the range of secure placements experienced by justice-involved youth. This broader definition is particularly important for adolescents and young adults, who may move between juvenile and adult systems and encounter varying institutional conditions that influence their development.

The total number of days was divided by the length of the recall period to calculate the proportion of time spent in correctional facilities. To ensure statistical appropriateness for the bounded nature of proportional data as outcomes and to enable unbounded linear modeling, this proportion was then logit-transformed $\left(\mathrm{logit}(p)=\ln\;\left(\frac{\;p}{1-p}\right)\right)$. This approach is commonly used in longitudinal and developmental research involving proportion data converted into log-odd ratios for a more symmetric and less skewed distribution with a higher likelihood of projecting a linear relationship between psychosocial maturity and time spent in correctional facilities than if the raw proportion data were utilized (48, 49). This transformation also facilitates interpretation of model coefficients: effects estimated on the logit scale represent multiplicative (percentage) changes in the odds of time spent in correctional facilities associated with a one-unit change in the predictor. Accordingly, coefficients can be exponentiated and interpreted as proportional increases or decreases in correctional exposure, providing clearer substantive interpretation than models estimated on the raw proportion scale. The resulting measure is referred to as *logit-transformed correctional time*.

#### Covariates

We included between-person covariates to account for potential confounding influences on time spent in secure facilities in the final model. These included sex (0 = female, 1 = male), race (two dummy variables for Black and Hispanic with White/Other as the reference group), study site (0 = Phoenix, 1 = Philadelphia), parental arrest history (0 = neither parent arrested, 1 = one parent arrested, 2 = both parents arrested), mental health symptoms, and intelligence. Mental health was measured using the 53-item Brief Symptom Inventory (50), assessing symptom distress in the past week on a 0 (not at all) to 4 (extremely) scale. Intelligence was assessed with the Wechsler Abbreviated Scale of Intelligence (51). This measure is normed for individuals aged 6–89.

### Analytic plan

To meaningfully estimate lagged and autoregressive effects, we standardized the measurement intervals by aggregating two 6-month periods into a single one-year interval. For psychosocial maturity, we used the measure from the end of each one-year period rather than averaging the two 6-month assessments, as the endpoint offers a temporally clearer reference for examining the influence of correctional exposure over the preceding year. All analyses were conducted using age-based data structure, aligning assessments by chronological age (e.g., age 15, 16, 17), rather than wave-based intervals. This approach improves developmental interpretation by aligning measurements with participants' age-specific changes, rather than arbitrary wave timing.

We used Random Intercept Cross-Lagged Panel Models [RI-CLPM; (52)] to separate between-person (trait-like) and within-person (dynamic) variance. This decomposition enabled us to isolate dynamic within-person processes over time without conflating them with stable individual differences, an improvement over traditional CLPM approaches (52, 53). Specifically, the random-intercept factors capture stable between-person differences in the overall level of each construct across the study period, reflecting trait-like variance that is constant over time. The autoregressive paths represent the degree to which an individual's within-person deviation at one time point carries over to the next assessment, capturing state-like fluctuations over time. The cross-lagged paths then test whether within-person deviations in one variable prospectively predict within-person deviations in the other variable at the next time point, net of each variable's own autoregressive carry-over. Throughout the paper, the terms “trait-like” and “state-like” refer to these between-person and within-person variance components, respectively.

Our analyses included two main parts. In Part I, we conducted a univariate model fitting for each construct. We compared several models: a random-intercept model with one-lag autoregressive effect (RI-AR1), a two-lag model (RI-AR2), and models with added growth components (linear and quadratic slopes). The quadratic slope was included to allow for non-linear change over time, as developmental trajectories often accelerate or decelerate across the observed period rather than changing at a constant rate. For models where both linear and quadratic growth components were tested, convergence failures producing negative residual variances (Heywood cases) were treated as evidence of overparameterization, and the affected model was excluded from further consideration. Model fit was assessed using *χ*^2^ tests, Root Mean Square Error of Approximation (RMSEA), Comparative Fit Index (CFI), and Bayesian Information Criterion (BIC). Models with non-significant RMSEA and *χ*^2^ tests, RMSEA below 0.05, and CFI above 0.90 were considered a good fit with the data (54). All models were estimated with maximum likelihood with robust standard errors (MLR). For nested models estimated using MLR, we applied the Satorra–Bentler scaled *χ*^2^ difference test (55, 56) for model selections. We also tested whether imposing time-invariant constraints on autoregressive effects would significantly worsen model fit. Testing time-invariant constraints on autoregressive effects serves to evaluate whether the within-person carry-over from one time point to the next is stable across age or varies over the study period. A time-invariant autoregressive effect assumes a single, constant parameter captures this carry-over at all ages, yielding a more parsimonious model; a time-variant specification estimates separate carry-over parameters at each lag. When the constrained model does not fit significantly worse, parsimony supports retaining the invariant structure, and the resulting single parameter estimate is carried forward into the bivariate model. When the constraint significantly worsens fit, the time-varying structure is retained to avoid model misspecification.

In Part II, we combined the best-fitting univariate models into a bivariate RI-CLPM that incorporated cross-lagged effects between psychosocial maturity and correctional time. We tested whether constraining cross-lagged paths to be time-invariant affected model fit. Time-invariant constraints on cross-lagged paths assume that the directional effect of psychosocial maturity on subsequent correctional time, and vice versa, is stable in magnitude across ages 15–22. Constraining these paths to equality across ages produces a single interpretable estimate per pathway and improves parsimony but requires empirical support. Last, we further examined whether effects were specific to particular domains of psychosocial maturity (i.e., temperance, responsibility, or perspective). Specifically, parallel bivariate RI-CLPMs were estimated separately for each psychosocial maturity component, allowing domain-specific within-person and cross-lagged associations with correctional time to be evaluated.

### Transparency and openness

We report all data exclusions, manipulations, measures, and sample size determinations. Models were estimated using Mplus 8.8 (57), with missing data handled via full information maximum likelihood. The analysis plan was not pre-registered. Replication code is available upon request. The present study involved secondary analysis of restricted-use, de-identified data obtained through the Inter-university Consortium for Political and Social Research (ICPSR) under a formal Data Use Agreement. It was approved by the Institutional Review Board at Georgia State University. All analyses were conducted in accordance with the terms of the restricted data agreement and applicable ethical guidelines.

## Results

Among the 1,336 participants, 80% spent time in correctional facilities during at least one of the ten follow-up periods. Specifically, 22.7% were confined during 1–2 periods, 26.7% during 3–5, 19.8% during 6–8, and 10% during 9 or all 10 periods. On average, participants spent 25% of each recall period on correctional facilities (SD = 38.25%; IQR = 43.30%).

Fewer observations were available for correctional time at each age year compared to psychosocial maturity. This discrepancy stems from the fact that psychosocial maturity was assessed at baseline, whereas correctional time was not. Aligning the data by age rather than study wave also contributed to uneven data coverage, particularly at ages 14, 15 and 23 and older, due to variability in age at baseline. These missing values reflect the age-based design rather than participant attrition. To ensure sufficient observations (*N* > 400), analyses were restricted to ages 16–22 for correctional time and 15–22 for psychosocial maturity. Cross-lagged models therefore span ages 15–22 for psychosocial maturity and 16–22 for correctional time, reflecting the absence of correctional data at baseline.

Descriptive statistics and age-specific correlations for logit-transformed correctional time and psychosocial maturity are summarized in the sTables 1 and 2 in Supplemental Material. As expected, both constructs demonstrated stronger correlations across adjacent years: *r*s = .49–.71 for correctional time and *r*s = .62–.71 for psychosocial maturity. Both also showed a general upward trend with age (see Supplementary Figure S1). Internal consistency across age years was good for global psychosocial maturity (*α* = .85–.90), temperance (*α* = .83–.87), and responsibility (*α* = .80–.84), while perspective demonstrated only adequate reliability (*α* = .72–.78).

 Table 1 presents bivariate correlations between psychosocial maturity and logit-transformed correctional time. Lagged associations, where psychosocial maturity at age t was correlated with correctional time at age *t* + 1, ranged from –.26 to –.15. Concurrent associations, measured at the same age, ranged from –.25 to –.11. These trends are illustrated in Supplementary Figure S2.

**Table 1:** Correlations between psychosocial maturity (PSM) and logit transformed correctional time (CT) and from ages 15 to 22.

Variables	CT15	CT16	CT17	CT18	CT19	CT20	CT21	CT22
PSM15	−0.14*	−0.25***	−0.21***	−0.18***	−0.16**	−0.18***	−0.20***	−0.15*
PSM16	−0.19**	−0.18***	−0.23***	−0.13***	−0.15***	−0.15***	−0.17***	−0.08*
PSM17	−0.21***	−0.23***	−0.25***	−0.17***	−0.18***	−0.17***	−0.17***	−0.15***
PSM18	−0.15*	−0.16***	−0.20***	−0.17***	−0.15***	−0.15***	−0.18***	−0.15***
PSM19	−0.17**	−0.16***	−0.16***	−0.12***	−0.11***	−0.13***	−0.18***	−0.12***
PSM20	−0.06	−0.13**	−0.14***	−0.12***	−0.10***	−0.12***	−0.17***	−0.11**
PSM21	−0.09	−0.12**	−0.10**	−0.10***	−0.10**	−0.10***	−0.16***	−0.13***
PSM22	0.1	−0.12*	−0.13***	−0.10**	−0.09**	−0.13***	−0.19***	−0.15***

* *p* < .05.

** *p* < .01.

*** *p* < .001.

### Part I: univariate models

**Figure 1:**
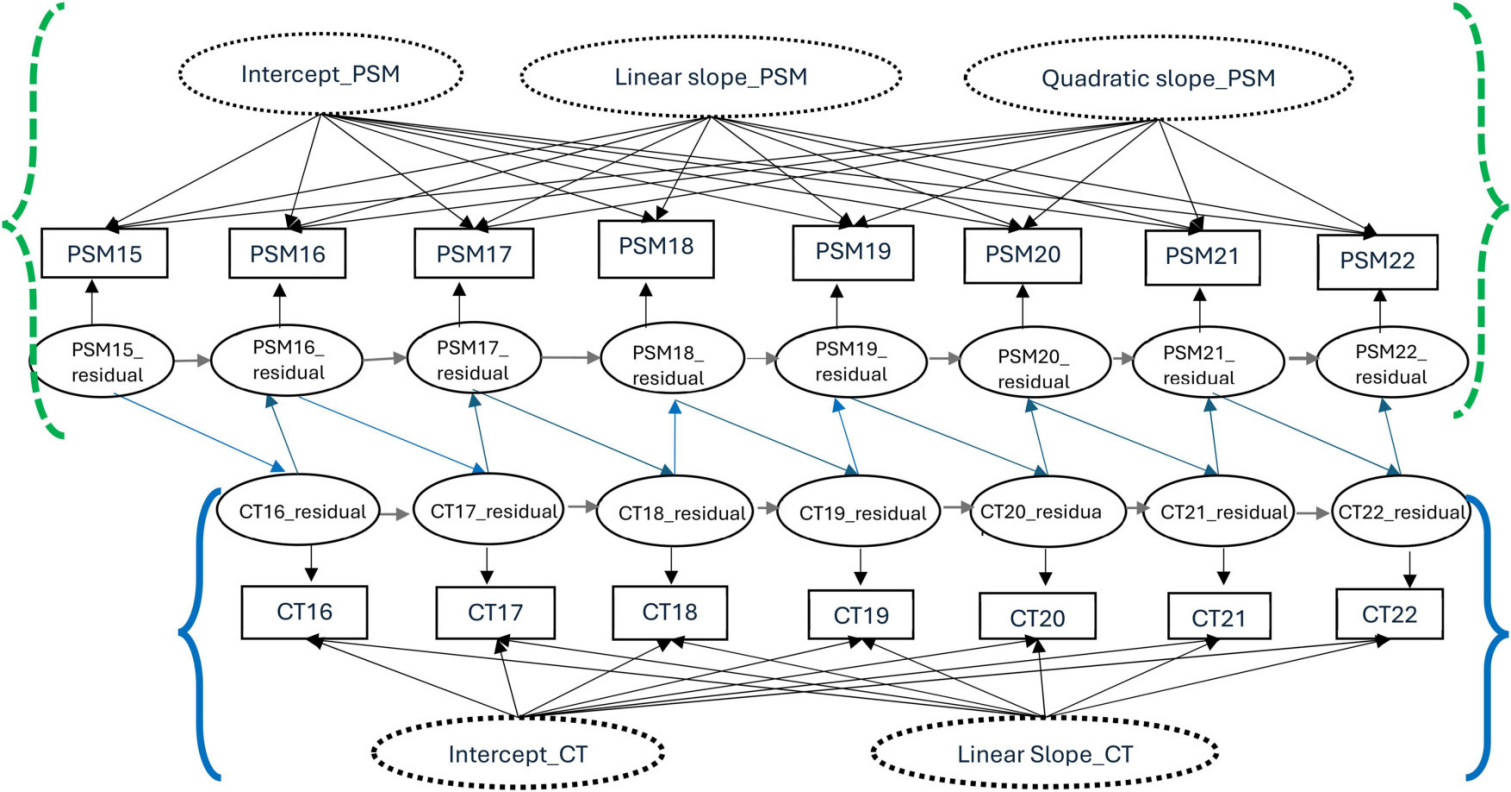
Illustration of the random-intercept cross-lagged panel model (RI-CLPM) with psychosocial maturity (PSM) and correctional time (CT) both having two-lagged autoregressive effects. The upper panel marked by dashed green bracket is the univariate model of PSM, and the bottom panel marked by solid blue bracket is the univariate model of correctional time. The association between all the growth factors (intercept, slopes; marked within dotted circles) of PSM and CT were omitted in display but was estimated.

For correctional time, models with the linear growth factor fit the data better (Models 3 and 4 in Table 2) as they had better RMSEA, CFI, and Model 3 had the lowest BIC of all models. Additionally, a comparison of Model 3 and 4 indicated that a one-lag autoregressive model did not fit the data significantly worse than the two-lag autoregressive model (Δ*χ*_5_^2^ = 9.60, *p* = 0.087). Growth models that included both linear and quadratic slopes failed to converge properly and produced a Heywood case, indicated by negative residual variances. This suggests overfitting or model misspecification due to the added complexity of the quadratic term. Overall, Model 3, a RI-AR1 growth model with linear slope, fit the data best. We further tested whether the autoregressive effects of correctional time were time-invariant. Models 3 and Model 5 were nested models and Satorra–Bentler *χ*^2^ difference test was significant (Δ*χ*_5_^2^ =  = 11.39, *p* = 0.044), suggesting models with time-variant AR1 effect fit the data significantly better than model with time-invariant AR1 effect. We proceed with RI-AR1 growth model with linear slope with time-variant AR effects for logit-transform correctional time (see the bottom illustration in Figure 1).

**Table 2:** Univariate models of logit-transform correctional time (*N* = 1,334, *T* = 7).

Model		BIC	*χ*^2^ test; (*p*)	RMSEA (*p*)	CFI
1	RI-AR1	44,949.88	*χ*_20_^2^ = 67.35; (0.000)	0.042 (0.869)	0.979
2	RI-AR2	44,958.43	*χ*_15_^2^ = 43.80; (0.000)	0.038 (0.931)	0.987
3	RI-AR1 growth model with linear slope	44,933.68	*χ*_17_^2^ = 32.83; (0.012)	0.026 (0.999)	0.993
4	RI-AR2 growth model with linear slope	44,957.58	*χ*_12_^2^ = 23.24; (0.026)	0.026 (0.994)	0.995
5	Model 3 with time-invariant AR1	44,912.14	*χ*_22_^2^ = 44.43; (0.003)	0.028 (1.000)	0.990

RI-AR1, random intercept model with one-lag autoregressive effect; RI-AR2, random intercept model with two-lag autoregressive effect; BIC, Bayesian Information Criterion; RMSEA, Root Mean Square Error of Approximation; CFI, Comparative Fit Index.

For psychosocial maturity, models with the linear and quadratic growth factor fit the data better (Models 3b and 4b in Table 3), indicated by better RMSEA, CFI, and non-significant *χ*^2^ test, than models having only linear slopes (Model 3a and 4a) or models without any growth factors (i.e., AR-only models, Models 1 and 2). Additionally, Models 3b and 4b were nested models and Satorra–Bentler *χ*^2^ difference test was not significant (Δ*χ*_6_^2^ = 6.12, *p* = 0.409**),** indicating the less complicated RI-AR1 growth model with linear and quadratic slopes (Model 3b) did not fit the data significantly worse than the more complicated RI-AR2 growth model with linear and quadratic slopes (Model 4b). In sum, Model 3b fits the data best.

**Table 3:** Univariate models of psychosocial maturity (*N* = 1,336, *T* = 8).

Model		BIC	*χ*^2^ test; (*p*)	RMSEA (*p*)	CFI
1	RI-AR1	2,897.48	*χ*_27_^2^ = 724.23; (0.000)	0.139 (0.000)	0.835
2	RI-AR2	2,829.57	*χ*_21_^2^ = 616.28; (0.000)	0.146 (0.000)	0.859
3	RI-AR1 growth models				
3a	linear slope	2,270.82	*χ*_24_^2^ = 91.94; (0.0000)	0.046 (0.728)	0.984
3b	linear, quadratic slope	2,236.12	*χ*_20_^2^ = 31.42; (0.0498)	0.021 (1.000)	0.997
4	RI-AR2 growth models				
4a	linear slope	2,283.28	*χ*_18_^2^ = 64.17; (0.0000)	0.044 (0.795)	0.989
4b	linear, quadratic slope	2,271.53	*χ*_14_^2^ = 26.47; (0.023)	0.026 (0.997)	0.997
5	Model 3b with time invariant AR1	2,197.71	*χ*_26_^2^ = 35.24; (0.1066)	0.016 (1.000)	0.998

RI-AR1, random intercept model with one-lag autoregressive effect; RI-AR2, random intercept model with two-lag autoregressive effect; BIC, Bayesian Information Criterion; RMSEA, Root Mean Square Error of Approximation; CFI, Comparative Fit Index.

We further tested whether the autoregressive effects of psychosocial maturity were time-invariant. Models 3b and Model 5 were nested models and Satorra–Bentler *χ*^2^ difference test was significant (Δ*χ*_6_^2^ = 4.200, *p* = 0.650), suggesting models with time-variant AR1 effect did not fit the data significantly better than model with time-invariant AR1 effect. Thus, we use Model 5 for psychosocial maturity (see the top illustration in Figure 1).

Parallel model comparisons for the three psychosocial maturity components, namely temperance, perspective, and responsibility, yielded substantively similar results, with the invariant AR1 growth model (Model 5) providing the best fit across all components (see Table 4).

**Table 4:** Univariate models of psychosocial maturity by core components (*N* = 1,336, *T* = 8).

Model		BIC	*χ*^2^ test; (*p*)	RMSEA (*p*)	CFI	Models 3b & 5 comparison
Temperance						
3a	RI-AR1 with linear slope	11,775.93	*χ*_24_^2^ = 45.32; (0.0053)	0.026 (1.000)	0.994	
3b	RI-AR1 with linear, quadratic slope	11,785.72	*χ*_20_^2^ = 27.76; (0.1153)	0.017 (1.000)	0.998	
5	Model 3b with time invariant AR1	11,747.27	*χ*_26_^2^ = 30.92; (0.2313)	0.012 (1.000)	0.999	Δχ_6_^2^ = 3.80; (0.74)
Responsibility						
3a	RI-AR1 with linear slope	6,393.50	*χ*_24_^2^ = 76.45; (0.0000)	0.040 (0.935)	0.981	
3b	RI-AR1 with linear, quadratic slope	6,365.12	*χ*_20_^2^ = 24.65; (0.2154)	0.013 (1.000)	0.998	
5	Model 3b with time invariant AR1	6,331.03	*χ*_26_^2^ = 32.36; (0.1814)	0.014 (1.000)	0.998	Δχ_6_^2^ = 7.70; (0.26)
Perspective						
3a	RI-AR1 with linear slope	8,401.209	*χ*_24_^2^ = 82.99; (0.0006)	0.030 (0.999)	0.989	
3b	RI-AR1 with linear, quadratic slope	8,387.34	*χ*_20_^2^ = 15.01; (0.7757)	0.017 (1.000)	0.998	
5	Model 3b with time invariant AR1	8,360.14	*χ*_26_^2^ = 28.36; (0.3410)	0.008 (1.000)	0.999	Δχ_6_^2^ = 12.39; (0.054)

RI-AR1, random intercept model with one-lag autoregressive effect; BIC, Bayesian Information Criterion; RMSEA, Root Mean Square Error of Approximation; CFI, Comparative Fit Index. Model comparison was conducted using Satorra–Bentler *χ*^2^ difference test.

### Part II: bivariate models

Correctional time at a given age reflects the time spent in secure facilities *during the age year*, while psychosocial maturity represents the developmental level reached *at the end assessment of that age year*. Therefore, it is temporally appropriate for psychosocial maturity at age *t* to predict correctional time at age *t* + 1 (lagged effect), and for correctional time at age *t* predicted psychosocial maturity at the end of age t, reflecting exposure accumulated during the preceding year (see Figure 1).

Guided by the best-fitting univariate models, we estimated a bivariate RI-CLPM including cross-lagged effects between psychosocial maturity and logit-transformed correctional time. All trait-like features (intercepts and slopes) were allowed to correlate freely. The model fits the data well (Δ*χ*_79_^2^ = 115.26, *p* = 0.005; CFI = 0.996, RMSEA = 0.019). A more parsimonious model with age-invariant cross-lagged effects also fit adequately (Δ*χ*_91_^2^ = 130.93, *p* = 0.004; CFI = 0.995, RMSEA = 0.018) and did not fit significantly worse than the age-variant model (Δ*χ*_12_^2^ = 15.65, *p* = 0.21**)**, so we retained the parsimonious age-invariant model. See Table 4 for results of cross-lagged effects.

In the final model, within-person effects indicated that deviations from each individual's expected developmental level of psychosocial maturity predicted subsequent deviations in correctional time: when individuals exhibited higher-than-expected psychosocial maturity relative to their own developmental trajectory, they experienced lower-than-expected correctional time in the following year. Conversely, greater-than-expected correctional time during a given year predicted lower-than-expected psychosocial maturity at the end of that year, relative to each individual's projected developmental course. These findings held when adjusting for sex, race, study site, mental health symptoms, and IQ with both cross-lagged effects being significant. The reciprocal effects were stable across ages 15–22. Moreover, at the between-person level, higher trait-like psychosocial maturity (i.e., random intercept differences) was strongly associated with lower overall correctional time across individuals (*B* = −0.673, SE = 0.111, *p* < 0.0005; *β* = −0.41), indicating that participants who had higher levels of psychosocial maturity in general tend to spend less time in correctional facilities.

Although time-invariance constraints were imposed on unstandardized cross-lagged paths, standardized coefficients (STDYX in Mplus) varied modestly across age years due to age-related changes in the variances of psychosocial maturity and correctional time. Across ages 15–22, standardized within-person cross-lagged effects were very small in magnitude: psychosocial maturity predicting subsequent correctional time averaged −0.045 (range: −0.058 to −0.038), whereas correctional time predicting subsequent psychosocial maturity averaged −0.035 (range: −0.040 to −0.029). Despite their small size, standardized effects were consistently negative in both directions and similar in magnitude across pathways.

#### Analyses on core components of psychosocial maturity

To identify which aspects of psychosocial maturity drive the reciprocal associations observed in the main model, we re-estimated bivariate Random Intercept Cross-Lagged Panel Models (RI-CLPMs) separately for each core component (i.e., temperance, responsibility, and perspective). Guided by the best-fitting univariate models, these component-level bivariate models adopted the same specification as the global psychosocial maturity model, as both the univariate growth structures of the psychosocial maturity components and correctional time were substantively similar. Accordingly, all models included correlated trait-like features (intercepts and slopes) and within-person cross-lagged effects.

For all three models, we first tested whether the cross-lagged effects could be constrained to be time-invariant. In each case, the constrained model did not fit significantly worse than the unconstrained model, supporting the use of time-invariant estimates (Δ*χ*_12_^2^ = 15.50, *p* = 0.22 for temperance, Δ*χ*_12_^2^ = 13.45, *p* = 0.34 for perspective, and Δ*χ*_12_^2^ = 16.30, *p* = 0.18 for responsibility.

Only temperance demonstrated significant reciprocal associations. Standardized within-person effects indicated that within-person deviations below an individual's expected temperance trajectory predicted subsequent deviations above expected correctional time (average *β* = −0.055, range: −0.068 to −0.047 across ages 15–22). Conversely, deviations above expected correctional time predicted subsequent deviations below expected temperance (average *β* = −0.056, range: −0.064 to −0.046). Although these standardized effects were very small in magnitude, they were consistently negative across ages and closely comparable across directions, indicating a stable bidirectional association between temperance and correctional time. Neither responsibility nor perspective showed significant effects in either direction. Detailed results are reported in Table 5.

**Table 5:** Age-invariant within-person cross-lagged effects (RI-CLPM), ages 15–22.

Variables	Within-person effects		Between-person association (random intercepts)
	Maturity at *t* predicting Correctional Time at *t* *+* *1*	Correctional Time at *t* predicting Maturity at *t*	Maturity and correctional time
	*B* (SE)	*B* (SE)	*B* (SE)
Total psychosocial maturity	−1.192** (0.444)	−0.001* (0.001)	−0.673*** (0.111)
Temperance	−0.799 ** (0.245)	−0.004** (0.001)	−0.665** (0.192)
Perspective	−0.355 (0.295)	−0.001 (0.001)	−0.686*** (0.165)
Responsibility	−0.124 (0.320)	0.001 (0.001)	−0.649*** (0.140)

Unstandardized coefficients B and standard error (SE) reported in the table. Cross-lagged paths constrained equal across waves (ages 15–22). Autoregressive paths for correctional time were estimated and not constrained to be constant across age, but results were omitted from the table; autoregressive paths for maturity were estimated and constrained equal, results omitted from the table.

* *p* < 0.05.

** *p* < 0.01.

*** *p* < 0.001.

## Discussion

This study examined the reciprocal relationship between psychosocial maturity and time in correctional facilities from adolescence through early adulthood using a within-person, longitudinal framework. Across ages 15–22, we found evidence of bidirectional associations: within-person decreases in psychosocial maturity relative to individuals' expected developmental trajectories predicted subsequent increases in correctional time, while greater-than-expected correctional time predicted slower-than-expected gains in psychosocial maturity. These reciprocal effects were statistically significant but small in magnitude and stable across age. Taken together, the findings indicate that psychosocial maturity is both a precursor to and a consequence of justice system involvement, underscoring the need to move beyond unidirectional models of incarceration effects.

A central contribution of this study lies in clarifying the directionality of associations between incarceration and psychosocial development. Prior studies have frequently emphasized the developmental harms of institutional exposure, often implicitly treating incarceration as an exogenous event (11, 15). While this work has been instrumental in documenting institutional deprivation effects, it has less often accounted for the possibility that preexisting developmental vulnerabilities increase the likelihood of incarceration in the first place. As a result, one-directional models risk conflating two processes: developmental suppression caused by incarceration and selection into incarceration driven by preexisting developmental vulnerabilities.

By estimating reciprocal within-person effects, the present study demonstrates that these processes operate simultaneously. When youth exhibited lower-than-expected psychosocial maturity relative to their own developmental trajectory, they were more likely to experience subsequent increases in correctional time, and that exposure, in turn, modestly constrains subsequent psychosocial development. Importantly, when only one direction is modeled, particularly incarceration predicting psychosocial maturity, unmodeled reverse pathways may inflate estimates of institutional harm by attributing variance associated with selection to deprivation. Our reciprocal framework helps address this attribution issue by partitioning variance attributable to both directions, yielding a more precise estimate of institutional effects that is neither dismissive of harm nor overstated. These findings reinforce calls for temporally sensitive designs that explicitly model bidirectional developmental processes [e.g. (20)].

The standardized within-person cross-lagged effects observed in this study were very small (−0.06 to −0.03), a finding that warrants careful interpretation. In the context of rigorous longitudinal models that isolate within-person change and adjust for stable between-person differences, effects of this size are not unexpected (52, 58). RI-CLPMs explicitly remove substantial variance attributable to trait-like stability, leaving only year-to-year deviations from an individual's typical developmental trajectory; as a result, standardized coefficients in these models reflect incremental rather than dramatic developmental shifts (59).

From a developmental psychopathology perspective, small effects can nonetheless be consequential when they recur across multiple years and involve developmentally salient exposures such as correctional confinement. Adolescence and early adulthood represent sensitive periods marked by heightened neurobiological plasticity and rapid maturation of self-regulatory and socioemotional systems (16, 17). During such periods, environmental inputs may exert amplified and lasting influence relative to other developmental stages. Developmental cascade models emphasize that modest perturbations in one domain can alter subsequent experiences and contexts, thereby shaping later development even when no single effect is large (60). In this framework, repeated within-person deviations may contribute to cumulative developmental processes by increasing exposure to constraining environments and limiting opportunities for normative skill acquisition (61, 62). While the present study does not directly test cumulative or accelerating effects, the observed pattern of stable, reciprocal associations across adolescence and early adulthood is consistent with cascade-based interpretations of developmental risk.

Our findings reinforce the importance of integrating the importation and deprivation models, which have too often been treated in isolation or competing explanations. The importation model (63) highlights dispositional vulnerabilities, such as poor impulse control and low future orientation, that elevate the risk of entering correctional settings. These traits have been consistently linked to delinquency and legal disadvantage (19, 26). Meanwhile, the deprivation model (64) emphasizes the suppressive effects of incarceration on psychosocial growth, particularly in highly controlled environments that limit autonomy and social learning. Supporting this, Thomas et al. (15) found that institutional exposure during adolescence predicted long-term deficits in emotional regulation and interpersonal functioning. Our results suggest these frameworks are not mutually exclusive but interact recursively: youth exhibiting lower-than-expected psychosocial maturity relative to their own developmental trajectory were more likely to experience subsequent correctional time, and youth with lower trait-like maturity also experienced greater overall confinement, creating a cycle of disadvantages and diminished growth.

Crucially, these processes interact recursively, and their consequences are particularly significant during the young adult period, which has received comparatively less empirical attention than adolescence despite representing a critical window for desistance. Moffitt's ensnarement hypothesis (10) specifically predicts that some individuals who would otherwise desist fail to do so because institutional contact during key developmental transitions creates barriers, such as disrupted self-regulation, limited prosocial role acquisition, and continued justice involvement, that are difficult to reverse. Our within-person findings directly support this mechanism: youth exhibiting lower-than-expected psychosocial maturity were more likely to experience subsequent correctional exposure, and that exposure in turn suppressed temperance gains, precisely the self-regulatory capacity most central to the behavioral transitions that support desistance. In this way, correctional time during a sensitive developmental window can “ensnare” youth not because of stable deficits, but because institutional contact interrupts psychosocial maturation that would otherwise support desistance.

Although the present study cannot directly assess life-course–persistent offending patterns due to the age range of the sample (15–22), the findings are consistent with ensnarement processes. At the within-person level, periods characterized by lower-than-expected psychosocial maturity were associated with subsequent increases in correctional time, and those exposures predicted slower-than-expected developmental gains. At the between-person level, youth with lower trait-like maturity also experienced greater overall correctional involvement. Thus, correctional time may not merely reflect preexisting immaturity but may be associated with modest drops from expected developmental growth that would otherwise facilitate normative transitions out of offending. Viewed through this lens, justice system involvement becomes not only a marker of risk but a mechanism through which developmental disadvantage can be reinforced, linking importation and deprivation processes within a broader cascade of cumulative developmental disruption.

Our findings also resonate with Sameroff's (65) transactional model, which posits that development emerges from continuous, bidirectional exchanges between an individual and their environment, with each shaping and being shaped by the other over time. In this framework, neither the child nor the environment is a fixed cause; rather, developmental outcomes are the product of ongoing transactions in which individual characteristics alter environmental responses, which in turn alter the individual. This maps naturally onto the reciprocal dynamics we observe: lower psychosocial maturity increases correctional exposure, and that exposure in turn constrains subsequent psychosocial development, with each transaction potentially compounding the next.

The transactional model is largely compatible with both the importation and deprivation frameworks in that it does not privilege dispositional or environmental explanations but instead treats their interaction as the unit of analysis. Where it extends our current approach is in its emphasis on the cumulative and transformative nature of these exchanges: each transaction is assumed to change both parties, meaning that the individual who re-enters the community after correctional exposure is not the same individual who entered, and the social environment that receives them has also been altered by their absence. Our RI-CLPM captures repeated exchanges across age but does not model how the nature or magnitude of these transactions may themselves change as a function of prior transactions, which is a limitation worth addressing in future work.

There are also points of potential incommensurability worth noting. The transactional model was developed primarily in the context of early parent-child relationships, where the environment (the caregiver) is a responsive agent capable of genuine behavioral change in response to the child. Correctional institutions are less obviously responsive in this sense; their organizational structure, staffing practices, and disciplinary cultures are relatively stable regardless of the characteristics of individual residents. This limits the degree to which the full transactional logic applies, at least at the institutional level. Future work could usefully examine whether specific relational components within correctional settings, such as officer-youth relationships or peer dynamics, function more transactionally, and whether the quality of those micro-level transactions moderates the developmental consequences of incarceration. Integrating the transactional model with life-course perspectives emphasizing turning points and social bonds (69) may also offer a more complete account of how correctional exposure reshapes developmental trajectories over time.

### Disaggregating psychosocial maturity

Another key substantive contribution of this study is the disaggregation of psychosocial maturity into its three core components (i.e., temperance, responsibility, and perspective) and revealed that the reciprocal relationship with correctional time was driven primarily by temperance. Within-person deviations below individuals' expected temperance trajectories predicted subsequent deviations above expected correctional time, and elevated correctional time predicted subsequent deviations below expected temperance. In contrast, responsibility and perspective showed no significant reciprocal associations. Consistent with our hypotheses, temperance demonstrated bidirectional within-person associations with correctional time, whereas responsibility and perspective did not. This pattern supports the proposition that behaviorally proximal regulatory capacities are more dynamically intertwined with justice system involvement than more distal, identity- or cognition-based components of psychosocial maturity. The null findings for responsibility and perspective therefore refine, rather than weaken, theoretical models of psychosocial development by highlighting domain-specific developmental sensitivity.

Temperance reflects behaviorally proximal self-regulatory capacities, including impulse control and suppression of aggression (5). Our finding aligns with prior research identifying self-regulatory skills as key predictors of delinquency and institutional adjustment (27, 28) and emphasizes the particular susceptibility of emotion regulation capacities to environmental stressors (35), such as those found in correctional settings. Environments characterized by surveillance and limited autonomy may reduce opportunities to practice self-control and exacerbate reactive tendencies, reinforcing this bidirectional dynamic, especially among youth already on antisocial trajectories. The bidirectional association observed for temperance thus reflects a self-reinforcing developmental process in which self-regulatory deficits and institutional exposure mutually exacerbate one another.

The absence of reciprocal effects for responsibility and perspective is theoretically informative. Responsibility, encompassing autonomy, self-reliance, and resistance to peer influence, reflects identity-based self-governance rather than moment-to-moment behavioral regulation. Its development depends on sustained opportunities for autonomous decision-making, role-taking, and identity formation (30, 31). It is possible that resistance to peer pressure, in particular, functions less as a malleable developmental outcome and more as a moderating factor that shaped how youth respond to environmental pressures rather than being directly altered by them.

Similarly, perspective, which encompasses future orientation and concern for others, likely draws on higher-order cognitive processes such as abstract reasoning, long-term planning, and prosocial motivation (34, 36). Although limited future orientation is associated with delinquency and persistence (35, 36), these capacities may be less behaviorally salient in the day-to-day structure of correctional settings. Rather than actively suppressing future orientation or empathy, these settings may simply fail to engage the developmental conditions such as autonomy, goal setting, and socially meaningful roles that are required for such capacities to evolve. Moreover, recent work suggests that short-term orientations may reflect adaptive responses to unstable or unpredictable environments rather than developmental deficits (38). Correctional settings, which emphasize short-term compliance and offer few opportunities for meaningful future planning, may neither promote nor substantially disrupt perspective, resulting in null reciprocal associations. As a result, perspective remains relatively stable during confinement, not because it is impervious to environmental influence, but because the environment lacks the complexity to affect it in either direction.

### Policy and intervention implications for developmentally informed justice systems

These findings have important implications for juvenile justice policy and intervention. Both trait-like differences and within-person fluctuations in temperance were associated with correctional time, suggesting that self-regulatory vulnerabilities function as both stable risk factors and dynamic developmental processes. At the between-person level, youth with lower trait-like temperance experienced greater overall correctional involvement, while at the within-person level, periods marked by lower-than-expected temperance were linked to subsequent increases in correctional time. Together, these patterns highlight the need for front-end interventions that identify and support youth with self-regulatory vulnerabilities before they become deeply involved in the justice system. Early identification and targeted support for temperance-related difficulties, including impulse control and emotional reactivity, through diversion programs, schools, or community-based settings may help reduce the likelihood of deeper system penetration.

At the same time, the finding that correctional time significantly suppresses temperance, albeit with small magnitude, reinforces calls for institutional reform to avoid compounding these developmental risks. Developmentally informed correctional settings that offer emotional regulation training, supportive adult relationships, and structured opportunities to practice self-control may help mitigate harm and promote growth. In contrast, the relative stability of responsibility and perspective suggests that these capacities may be less amenable to short-term environmental interventions and may require more sustained, developmentally sequenced support. More broadly, the results argue for a dual-focus policy strategy: prevention before system entry through early psychosocial screening and skill-building, and protection during custody through targeted, evidence-informed programming focused on the most developmentally sensitive domains. Treating psychosocial maturity as both a risk factor and an outcome shifts the focus from whether incarceration is harmful to how and for whom it is developmentally consequential.

Several limitations should be considered when interpreting these findings. First, although the use of Random Intercept Cross-Lagged Panel Models strengthens causal inference by separating within-person dynamics from stable between-person differences, the analyses remain observational and cannot fully rule out unmeasured time-varying confounders. Second, while our measure of correctional time offers a comprehensive index of institutional exposure across different facility types, it does not capture qualitative aspects of these environments (e.g., therapeutic services, peer dynamics) that may moderate developmental outcomes. Third, our sample, while diverse and representative of serious juvenile offenders, includes only youth from two geographic regions and may not generalize to lower-risk populations or non-urban contexts, and may not readily transfer to other juvenile justice systems. Moreover, the majority of the sample were male and the generalization to female should be made with caution. Fourth, psychosocial maturity was assessed via self-report, which may introduce shared method variance or reporting biases; however, the longitudinal within-person design reduces the likelihood that stable reporting tendencies account for the observed cross-lagged associations. Finally, while statistically significant, the small effect sizes caution against overstating practical impact and highlight the cumulative nature of developmental risk.

## Conclusions

By modeling psychosocial maturity and correctional time as reciprocally linked developmental processes, this study advances understanding of how justice system involvement intersects with adolescent development. The findings show that within-person fluctuations in psychosocial immaturity were linked to subsequent correctional time, and correctional time was associated with modest deviations in subsequent developmental growth, while other components of maturity operate through distinct pathways. These results refine theoretical models of importation, deprivation, and ensnarement, and underscore the importance of developmentally informed policies that address both who becomes incarcerated and how incarceration shapes developmental trajectories. A priority for future work is identifying which features of correctional environments, such as emotional regulation programming and supportive officer-youth relationships, can buffer the suppressive effects on self-regulatory development documented here, moving the field from documenting that incarceration disrupts development toward specifying the conditions under which it need not.

## Data Availability

The datasets presented in this article are not readily available because the present study involved secondary analysis of restricted-use, de-identified data obtained through the Inter-university Consortium for Political and Social Research (ICPSR) under a formal Data Use Agreement. Data cannot be shared by the authors of this paper but need to be requested separately via ICPSR. Requests to access the datasets should be directed to ICPSR-help@umich.edu.
