# Supplementary material for: Psychosocial maturity and institutional confinement from ages 15–22: a longitudinal reciprocal model
Source: Front Child Adolesc Psychiatry. 2026 Jun 10;5:1809393. doi: 10.3389/frcha.2026.1809393 (PMC13290967; doi:10.3389/frcha.2026.1809393)
Supplement: Supplementary file 1 [file Table1.docx]

**Supplemental material**

**Results**

Descriptive statistics for the Logit-transformed correctional time and lagged correlations are summarized in sTable 1. Correlations for correctional time were stronger between adjacent age years, ranging from 0.49 to 0.71, than between more distant years. Descriptive statistics for psychosocial maturity are reported in sTable 2 along with the Cronbach’s α for global and core components of psychosocial maturity. Correlations for psychosocial maturity at different age years followed a similar pattern as that of correctional time, with high correlations between adjacent years ranging from 0.62 to 0.75.

sFigure 1 display trends in correctional time and psychosocial maturity, respectively. Participants' correctional time increased during adolescence and plateaued around 20 years old, and psychosocial maturity increased with age, albeit with modest change given the 4-point scale.

**sTable 1** Descriptive Statistics and Pearson’s Correlations of **Logit-transformed Correctional Time (Logit Time)** from Age 15 to 24.

|  | 15 | 16 | 17 | 18 | 19 | 20 | 21 | 22 |
| --- | --- | --- | --- | --- | --- | --- | --- | --- |
| Logit Time15 | 1 |  |  |  |  |  |  |  |
| Logit Time16 | 0.49 | 1 |  |  |  |  |  |  |
| Logit Time17 | 0.42 | 0.56 | 1 |  |  |  |  |  |
| Logit Time18 | 0.39 | 0.40 | 0.55 | 1 |  |  |  |  |
| Logit Time19 | 0.42 | 0.37 | 0.41 | 0.65 | 1 |  |  |  |
| Logit Time20 | 0.41 | 0.34 | 0.42 | 0.51 | 0.62 | 1 |  |  |
| Logit Time21 | 0.31 | 0.28 | 0.32 | 0.44 | 0.52 | 0.65 | 1 |  |
| Logit Time22 | 0.22 | 0.29 | 0.33 | 0.43 | 0.47 | 0.56 | 0.71 | 1 |
|  |  |  |  |  |  |  |  |  |
| N | 271 | 589 | 982 | 1253 | 1230 | 1195 | 1150 | 1005 |
| Mean | -9.33 | -8.56 | -8.37 | -8.55 | -8.23 | -7.76 | -7.81 | -7.84 |
| Median | -13.82 | -13.82 | -13.82 | -13.82 | -13.82 | -13.82 | -13.82 | -13.82 |
| SD | 5.79 | 6.04 | 6.15 | 6.1 | 6.22 | 6.27 | 6.29 | 6.31 |
| Min | -13.82 | -13.82 | -13.82 | -13.82 | -13.82 | -13.82 | -13.82 | -13.82 |
| Max | 0.05 | 0.09 | 0.17 | 0.11 | 0.24 | 0.19 | 0.15 | 0.09 |
| IQR | 11.25 | 11.79 | 12.34 | 12.21 | 12.75 | 12.96 | 12.93 | 12.99 |

*Note.* All correlations shown were significant with *p* < 0.05. SD = standard deviation.

**sTable 2.** Descriptive Statistics and Pearson’s Correlations of Psychosocial Maturity (PSM) from Age 15 to 24.

|  | 15 | 16 | 17 | 18 | 19 | 20 | 21 | 22 |
| --- | --- | --- | --- | --- | --- | --- | --- | --- |
| PSM15 | 1 |  |  |  |  |  |  |  |
| PSM16 | 0.62 | 1 |  |  |  |  |  |  |
| PSM17 | 0.54 | 0.63 | 1 |  |  |  |  |  |
| PSM18 | 0.53 | 0.58 | 0.67 | 1 |  |  |  |  |
| PSM19 | 0.45 | 0.56 | 0.6 | 0.69 | 1 |  |  |  |
| PSM20 | 0.5 | 0.55 | 0.57 | 0.68 | 0.73 | 1 |  |  |
| PSM21 | 0.49 | 0.54 | 0.57 | 0.64 | 0.66 | 0.74 | 1 |  |
| PSM22 | 0.4 | 0.49 | 0.51 | 0.6 | 0.61 | 0.68 | 0.74 | 1 |
|  |  |  |  |  |  |  |  |  |
| N | 413 | 813 | 1202 | 1271 | 1230 | 1194 | 1149 | 1003 |
| Mean | 2.7 | 2.74 | 2.8 | 2.86 | 2.91 | 2.96 | 2.96 | 3 |
| Median | 2.69 | 2.72 | 2.78 | 2.85 | 2.89 | 2.94 | 2.95 | 2.99 |
| SD | 0.37 | 0.38 | 0.37 | 0.37 | 0.38 | 0.38 | 0.38 | 0.37 |
| Min | 1.66 | 1.69 | 1.78 | 1.74 | 1.65 | 1.61 | 1.66 | 1.99 |
| Max | 3.68 | 3.75 | 3.89 | 3.94 | 3.9 | 3.96 | 3.95 | 3.87 |
| IQR | 0.51 | 0.56 | 0.52 | 0.53 | 0.53 | 0.56 | 0.55 | 0.53 |
| Cronbach’s α |  |  |  |  |  |  |  |  |
| Total PSM | 0.85 | 0.86 | 0.87 | 0.88 | 0.89 | 0.90 | 0.90 | 0.89 |
| Temperance | 0.83 | 0.85 | 0.86 | 0.87 | 0.87 | 0.87 | 0.87 | 0.87 |
| Perspective | 0.78 | 0.78 | 0.76 | 0.72 | 0.72 | 0.76 | 0.77 | 0.76 |
| Responsibility | 0.82 | 0.80 | 0.82 | 0.82 | 0.84 | 0.84 | 0.84 | 0.84 |

*Note.* All correlations shown were significant with *p* < 0.05. SD = standard deviation.


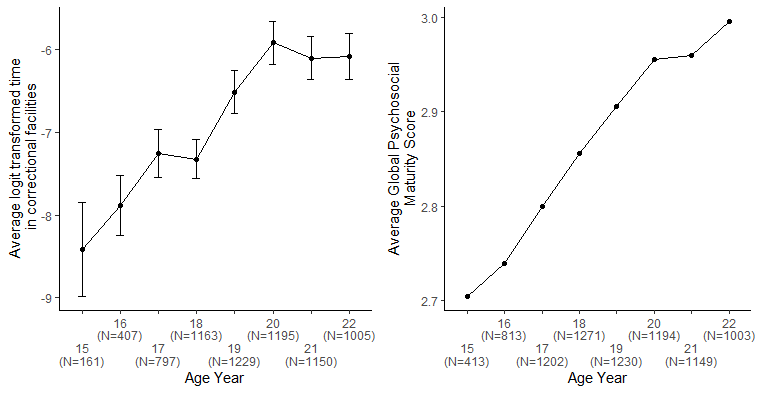


**sFigure 1.** Average proportion (logit-transformed) of recall period spent in correctional facilities (left) and average global psychosocial maturity (right) by age years

**
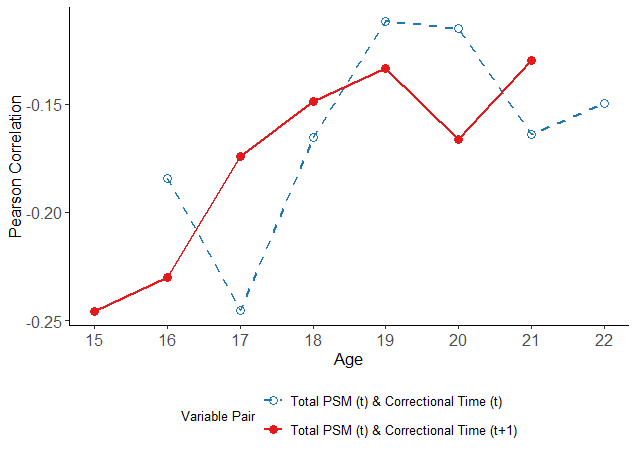
**

**sFigure 2**. Correlation coefficients between PSM and logit transformed proportion of recall period in correlation facility concurrently and subsequently by age groups
